# Label-Free Bioelectrochemical Methods for Evaluation of Anticancer Drug Effects at a Molecular Level

**DOI:** 10.3390/s20071812

**Published:** 2020-03-25

**Authors:** Francesco Tadini-Buoninsegni, Ilaria Palchetti

**Affiliations:** Department of Chemistry “Ugo Schiff”, University of Florence, 50019 Sesto Fiorentino, Italy

**Keywords:** anticancer drugs, bioelectrochemistry, impedance-based methods, solid supported membranes, DNA-based biosensor, drug–protein interactions, drug–DNA interactions, drug–cell interactions

## Abstract

Cancer is a multifactorial family of diseases that is still a leading cause of death worldwide. More than 100 different types of cancer affecting over 60 human organs are known. Chemotherapy plays a central role for treating cancer. The development of new anticancer drugs or new uses for existing drugs is an exciting and increasing research area. This is particularly important since drug resistance and side effects can limit the efficacy of the chemotherapy. Thus, there is a need for multiplexed, cost-effective, rapid, and novel screening methods that can help to elucidate the mechanism of the action of anticancer drugs and the identification of novel drug candidates. This review focuses on different label-free bioelectrochemical approaches, in particular, impedance-based methods, the solid supported membranes technique, and the DNA-based electrochemical sensor, that can be used to evaluate the effects of anticancer drugs on nucleic acids, membrane transporters, and living cells. Some relevant examples of anticancer drug interactions are presented which demonstrate the usefulness of such methods for the characterization of the mechanism of action of anticancer drugs that are targeted against various biomolecules.

## 1. Introduction

Cancer is a family of complex diseases that can start in almost any organ or tissue of the body. Cancer is the second cause of mortality in economically developed countries, accounting for an estimated 9.6 million deaths, in 2018 [1]. Lung, prostate, colorectal, stomach, and liver cancers are the most common types of cancer among men, while breast, colorectal, lung, cervical, and thyroid cancers are the most common in women [1]. It has been estimated that by the year 2050, 27 million new cancer cases will be diagnosed [2]. Currently, cancer treatment involves surgery, chemotherapy, radiotherapy, immunotherapy, hormone therapy, and other targeted therapies, with chemotherapy playing a central role. Anticancer drugs that treat different types of tumors are available, and can be used as a single agent or in combination with a wide range of other drugs. Traditional anticancer drugs cause several undesired side effects and despite the fact that cancer initially responds to chemotherapy, cancer cells can gain resistance and they can adapt to survive. Furthermore, while primary cancer tumors are treatable with chemo- and radiotherapies, metastatic cancer is difficult to treat with available chemotherapies [3]. 

Hence, a pressing demand has directed researchers towards the development of rapid and simple techniques for the investigation of interactions of cancer cells with drugs at different molecular levels and at different stages of the disease. Currently, following the 3R principle: “reduction, replacement, and refinement” of animal use [4], anticancer screening tests are performed by using in silico and in vitro approaches, selecting the most reliable candidates before evaluating the behavior in vivo. Many in vitro advanced methods can be used to study drug interactions in a cell population or in a tissue both in a label or label-free approach. In terms of time, cost, and ease of the analysis, label-free methods are preferable in both high-throughput and low-throughput screening tests. High-throughput tests are devoted to monitor drug–cell interactions in order to elucidate pathways and to characterize metabolic, pharmacokinetic, and toxicological data about new drugs [5], whereas low-throughput tests which are based on individual receptors are useful to define kinetics and thermodynamics of the drug–receptor interactions. Some recent review papers describe the main important in vitro tests for anticancer drug screening and we ask the reader to refer to these papers for a detailed description of the different methods for in silico and in vitro assays [3,4].

This present paper focuses on the use of bioelectrochemistry for evaluating the effects of anticancer drugs at the molecular level. Three different label-free bioelectrochemical strategies are presented, i.e., impedance-based methods, electrical technique which makes use of a solid supported membrane, and electrochemical nucleic acid-based sensors. These experimental methods have been used to investigate the mechanism of action of certain anticancer drugs and their effects on living cells, on membrane transport proteins, and on nucleic acids. Some relevant examples of anticancer drug interactions with DNA, membrane transporters, and cells are presented. The reported data qualify such bioelectrochemical approaches as robust and simple assays that could represent attractive analytical tools in drug development and evaluation.

## 2. Impedance-Based Methods for Cell Monitoring

Impedance-based cell monitoring, pioneered by Giaever and Keese, in the 1980s [6], has become a label-free, robust, minimally-invasive, nondestructive, cost-effective technology for real-time anticancer drug screening [7,8,9] and cytotoxicity evaluation [10], which can be easily multiplexed [11]. Compact instrumentations for multiplexed analysis are commercially available such as the xCELLigence® tools from ACEA Biosciences Inc. (San Diego, CA, USA), recently acquired by Agilent, or ECIS® from Applied BioPhysics (Troy, NY, USA).

**Figure 1:**
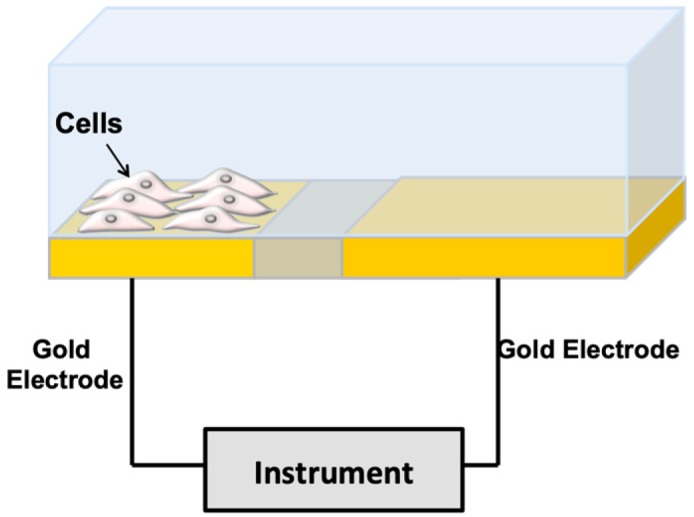
Impedance-based method for cell monitoring. The cells are deposited over gold microelectrodes in a culture medium. The resistance components are due to the current flow under the cells and the resistance is due to the current flow between the cells. The capacitive component is due to current flow through the cell membranes (from [10] with permission).

When cells are immobilized on the surface of microelectrodes, they impede electrical current flowing due to the interference of the anchored insulating bilayer lipid membrane over the microelectrode surface (Figure 1). In impedance spectroscopy, a voltage and a small sinusoidal alternating voltage (E_ac_) perturbation (5 to 10 mV amplitude) are applied between the working electrode and the reference electrode of an electrochemical cell. The impedance is monitored versus time. Impedance depends on conductance, capacitance, and inductance of an electrochemical system. Due to their effect on the electrical current flow, cells can be modeled by basic impedance elements including capacitors (plasma membrane) and resistors (the combination of all ion channels for the exchange of ions across the membrane) [8]. The growth of a cell culture can be monitored by evaluating the electrode impedance, which changes with cell surface coverage. Moreover, as a reaction to a toxicant, impedance dynamically fluctuates with cell motility or in response to cellular metabolism. The death of the cells causes their detachment from the electrode surface. This phenomenon causes a drop in the recorded impedance, which indicates a reduction in the number of viable cells. Thus, impedimetric measurements provide a continuous, real-time, label-free approach of cell count, cell morphology, cell motility and viability. 

**Figure 2:**
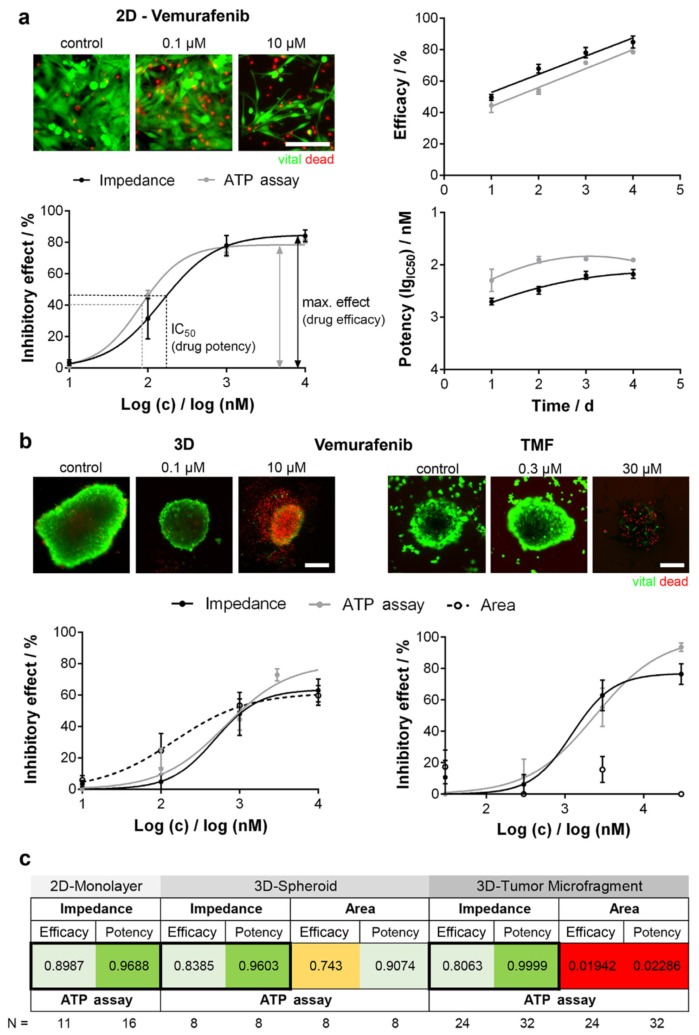
Comparison of potency and efficacy of Vemurafenib (a BRAF inhibitor) using different BRAF mutated melanoma models. (**a**) Normalized concentration-response curves of a two-dimensional (2D) cell line (left). Potency and efficacy plotted over time (right); (**b**) Concentration-response curves in three-dimensional (3D) cell cultures (left) and tumor microfragments (TMF, right) obtained with EIS, ATP assay, and size detection (cross-section area). Viability staining visualizes drug effects. Scale bar 200 µm; (**c**) Correlation analysis. Highest correlation (black framed boxes). Reprinted with permission from [19].

Impedimetric assays were performed to investigate the effects of anticancer drugs on different tumor-derived cell lines. Doxorubicin (DOX), an anthracycline-based antibiotic widely used in the treatment of a broad range of solid tumors, as well as acute leukemia and malignant lymphoma, has been used in many reports as a model molecule for studying the effect of dose or time dependency on a specific cell population density [12,13,14], as well as to distinguish cell models of acquired drug resistance [15]. Table 1 summarizes some examples of anticancer drugs that have been evaluated by impedance-based methods. Single cell or few cell populations [16], large cell populations, and three-dimensional (3D) cell aggregates, such as cell spheroids [17,18], have been tested. A recent report by Seidel et al. [19] focused on the evaluation of derived two-dimensional (2D) and 3D cell cultures with original patient undissociated melanoma tissues in order to develop cell model data of combined targeted cancer therapy and to transfer these data to an in vivo situation [19]. Figure 2 shows the correlation of chemosensitivity and drug kinetics obtained by cell impedance and by standard ATP assay on the different cell cultures. The drug potency (IC_50_ value) and efficacy (maximum inhibitory effect) were determined and plotted over time to reveal drug activity. Concentration-response curves showed a similar trend for ATP assay and impedance data. No significant difference was observed between efficacy and IC_50_ values evaluated by the two methods (Figure 2a–c). Moreover, the viability staining (Figure 2b) shows a decrease of viable cells and an increase of dead cells in a concentration-dependent manner. As reported in Figure 2b,c, reduced comparability of the ATP assay and EIS data with 3D culture size is shown for the tumor microfragments chemosensitivity analysis. Even with high drug effects that caused complete cell destruction, 3D structures did not change size, probably because of the substantial fraction of extracellular matrix in patient-derived non-dissociated tissue. This result was also shown in the concentration-response curves, where ATP data and impedance data showed a similar trend, but regression was not possible for size measurement.

Thus, substantial differences in drug response patterns between artificial in vitro and organotypic ex vivo cell cultures highlight the importance of in vivo-like cell models in drug development. New classes of personalized detection devices to study drug-induced cellular events and optimize the drug treatment of a patient during chemotherapy have opened new avenues in personalized medicine. Impedance-based cell analysis combined with real-time imaging could represent a useful technique for identification of cancer cells at different stages and their interaction with drugs during the disease.

Impedance-based measurements can also be performed on a flow of cell suspension. In impedance flow cytometry, the electrical properties of single cells can be measured [11,20]. The cells are polarized using an alternate current electric field. At low frequencies, the cell membrane impedes the current flow and the measurement of current amplitude indicates the cell volume or size. At intermediate frequencies, the capacitance of the suspension is decreased as the amount of plasma membrane polarization decreases. Measurements in this range of frequency are correlated to plasma membrane properties. At high frequencies, the plasma membranes are minimally polarized, and information regarding the dielectric properties of the cell interior can be obtained. Impedance-based flow cytometry analyzes a single cell instead of a population of cells. Portable, easy to use instrumentations are commercially available, such as the Amphasys’ Ampha^TM^ Z32 impedance flow cytometer.

**Table 1:** Some examples of anticancer drugs studied by impedance-based methods in cancer cell lines.

Anticancer Drug	Tumor Derived Cell Lines	Observed Effect	Specific Comments	Comparison Biochemical Assay	Ref
Carboplatin, Paclitaxel	Breast cancer, melanoma and human prostate cancer cells	Cell viability	Microfluidic platform. Dynamic delivery of the drug to cancer cells seeded in a chamber containing interdigitated microelectrodes.	MTT assay	[21]
Cisplatin	Oral cancer	Cell viability	High concentration of nicotine exhibited inhibitory effect on 20 μM cisplatin-induced apoptosis.	-	[22]
Cisplatin	Brest cancer	Cell attachment, spreading and drug-induced apoptosis	Time dependent behavior.	Morphological analysis	[16]
Cisplatin	Esophageal cancer	Cell morphology	Morphology changes of cells adhesion, spreading, and proliferation can be detected by impedimetric analysis.	Fluorescence imaging	[23]
Doxorubicin	Neuroblastoma and glioblastoma	Cell viability	Time dependent IC_50_. IC_50_ at 48 h for neuroblastoma cells: 1.77 nM IC_50_ at 48 h for glioblastoma cells: 4.04 nM	Tunel assay, Flow cytometry	[17]
Doxorubicin	Laryngopharynx cancer	Cell viability	Microfluidic platform enabling both electrochemical and optical detection.	Fluorescence-based cytotoxicity assay (annexin V/propidium iodide end point staining).	[13]
Doxorubicin	Breast cancer	Cell morphology	Drug resistant breast cancer cells have been differentiated from their parental cells based on their dielectric properties. Drug response at different stages of the disease is described.	Fluorescence microscopy	[15]
Etoposide	Neuroblastoma and glioblastoma	Cell viability	Time dependent IC_50_. IC_50_ at 48 h for neuroblastoma cells: 3.83 nM	Tunel assay, flow cytometry	[17]
Fluorouracil	Cancer microtissue spheroids	Cell viability	Evaluation of a multiplexed EIS platform analysis in a microfluidic setting.	-	[24]
Nicotine, Antrodia Camphorata ext.	Different cell lines	Cell morphology	-	SEM imaging	[25]
Vemurafenib and other MAPK-targeting therapeutics	Melanoma	Cell viability	Comparability of chemosensitivity performed by correlation analysis, showing that impedance and ATP assay data were highly correlative (0.8 < r^2^ < 1.0)	ATP assay	[19]
Vincristine	Neuroblastoma and glioblastoma	Cell viability	IC_50_ at 48 h for neuroblastoma cells (3D cultures): 1.16 nM IC_50_ at 48 h for glioblastoma cells (3D cultures): 1.54 nM	Tunel assay, flow cytometry	[17]
ZD6474	Breast cancer	Cell viability	Time and drug concentration dependent behavior	MTT assay	[26]

## 3. Solid Supported Membranes for Functional Analysis of Membrane Transporters

An electrical method based on a model membrane system, the so-called solid supported membrane (SSM), has been widely used to investigate membrane transport proteins. Membrane transporters move charged substrates across a biological membrane while going through their transport cycle and are responsible for the generation and maintenance of ion gradients, the transport of metabolites and signaling molecules, the uptake of nutrients, and the disposal of toxic compounds. Membrane transporter dysfunction is related to various disease states, which include cardiovascular, neurological, metabolic, and inflammatory diseases. Because of their relevance to a wide range of cellular functions, membrane transporters are drug targets of increasing importance to the pharmaceutical industry.

The SSM method allows label-free direct electrical measurements of charge movement across the membrane transporter immobilized on the SSM surface, yielding useful information about the transport activity and protein function. Thanks to its robustness and potential for automation, the SSM technique can be conveniently used to evaluate pharmacological agents affecting membrane transport proteins.

**Figure 3:**
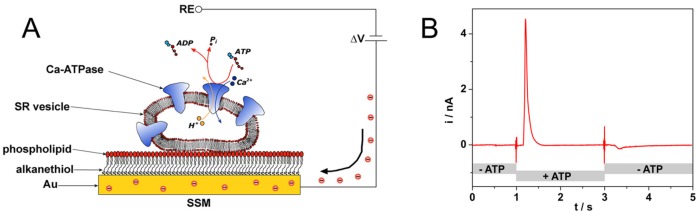
(**A**) Schematic diagram of a sarcoplasmic reticulum (SR) vesicle containing Ca-ATPase adsorbed to an solid supported membrane (SSM) and subjected to an ATP concentration jump (not drawn to scale). If the ATP jump induces a net charge movement across the protein, a compensating current is generated along the external circuit (the red spheres represent electrons) to keep constant the potential difference ΔV across the whole metal/solution interface. RE is the reference electrode. Reprinted from [36] with permission from Elsevier; (**B**) Current signal after an ATP concentration jump on SR vesicles incorporating Ca^2+^-ATPase. The ATP jump induces a current transient (current amplitude of 4.5 nA at ~1.2 s) that is related to charge movement across the protein. ATP removal determines a small signal of negative amplitude (at ~3.3 s), which is due to the discharge of SSM capacitance. Solution exchange into the cuvette containing the SSM sensor is controlled by electromechanical valve opening/closing (at 1 s and 3 s). Reprinted by permission from [33]. Copyright 2016 Springer Nature.

SSMs are usually formed by a hybrid alkanethiol/phospholipid bilayer supported by a gold electrode [27,28,29,30,31]. To obtain the SSM, the gold surface is covered by a densely packed alkanethiol monolayer, typically an octadecanethiol monolayer. A phospholipid monolayer is then self-assembled on the gold-supported thiol layer, so that the alkyl chains of the phospholipid are in contact with those of the alkanethiol (Figure 3A) [29,32]. Membrane fragments and proteoliposomes incorporating the membrane transporter are adsorbed to the SSM surface (Figure 3A), and the protein is, then, activated by a substrate concentration jump. Following protein activation, a current signal is measured which is related to charge movement across the membrane transporter [33]. As an example, an ATP concentration jump on sarcoplasmic reticulum (SR) vesicles containing Ca^2+^-ATPase induces a current signal (Figure 3B) that is connected with ATP-dependent Ca^2+^ translocation by the enzyme [34]. We point out that the charge movement across the transport protein is transmitted to the measuring circuit via the SSM capacitance and the resulting capacitive current is recorded as a function of time (current transient) [35,36]. Typical current amplitudes range from 0.1 to 10 nA.

The SSM method has been employed in basic research to study various membrane transport proteins that belong to different families. Most transporters studied are P-type ATPases (ion pumps) [36] and secondary active transporters [37,38,39,40,41]. In the case of P-type ATPases, which are a very important class of drug targets [42], SSM-based current measurements have been performed to investigate the transport mechanism of Na^+^,K^+^-ATPase [32,43], SR Ca^2+^-ATPase [34,44], H^+^,K^+^-ATPase [45], Cu^+^-ATPases [46,47], and very recently a P4-ATPase phospholipid flippase [48]. 

It is worth noting that the SSM electrode combined with robotized instrumentation is an attractive tool for screening applications in drug discovery [45,49]. Commercial semiautomatic and automatic analysis systems for SSM-based electrical measurements are available. A single channel semi-automated analysis device (SURFE^2^R N1, Nanion Technologies, Munich, Germany) is currently used in academia for basic research purposes. To address the requirements of the pharmaceutical industry for a higher throughput and a lower reagent consumption, SSM-based devices capable of performing fully automated measurements have been introduced. The SURFE^2^R 96SE device (Nanion Technologies) is especially suitable for drug screening purposes. This instrument is able to measure electrical currents simultaneously from 96 individual SSM sensors in a standard 96-well plate, allowing determination of the dose dependence of 100 compounds in less than 30 min [50].

### Anticancer Drug–Protein Interactions Monitored on SSMs

The SSM method has been employed to evaluate the effects of compounds of pharmacological interest on the activity of various membrane transporters. Some relevant examples of drug–protein interactions investigated by the SSM technique are here reviewed.

**Figure 4:**
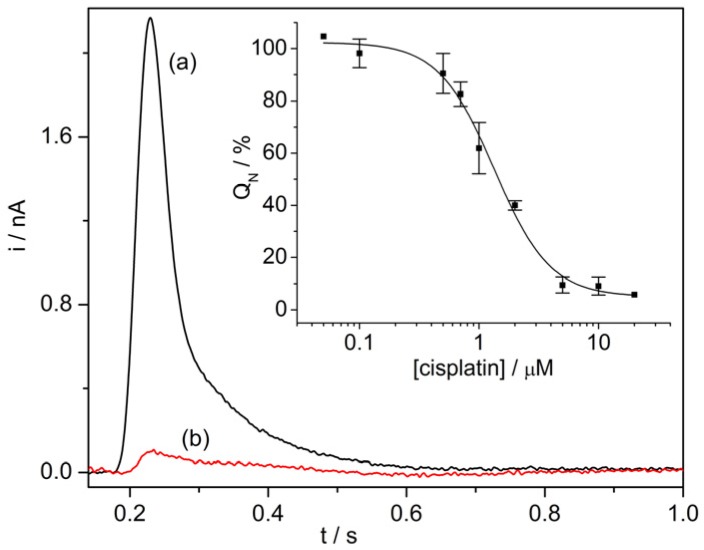
SR Ca^2+^-ATPase current signals induced by 100 µM ATP concentration jumps in the presence of 10 µM Ca^2+^ and in the absence (control measurement, black line, a) or in the presence of 5 µM cisplatin (red line, b). Inset: Normalized charges (Q_N_) related to ATP concentration jumps as a function of cisplatin concentration. The charges were normalized with reference to the maximum charge attained in the absence of cisplatin (control measurement). The solid line represents the fitting curve to the ATP-induced charges (IC_50_ = 1.3 ± 0.1 µM). The error bars represent S.E. of three independent measurements. Reproduced by permission of The Royal Society of Chemistry from [53].

SSM-based current measurements were carried out to study the effects of anticancer drugs on two P-type ATPases, i.e., Na^+^,K^+^-ATPase and SR Ca^2+^-ATPase. The Na^+^,K^+^-ATPase is found in the plasma membrane of animal cells. This enzyme transports three Na^+^ ions out of and two K^+^ ions into the cell using energy from ATP hydrolysis, thereby generating electrochemical potential gradients of Na^+^ and K^+^ ions that are crucial for a number of cell functions [51]. The Ca^2+^-ATPase that is present in the SR of muscle cells hydrolyzes one ATP molecule to transport two Ca^2+^ ions against their electrochemical potential gradient from the cytoplasm to the SR lumen, thereby inducing muscle relaxation [52]. The interaction of cisplatin with these two prominent enzymes was recently characterized [53]. Cisplatin, a well-established platinum-containing drug, is used to treat several human cancers [54]. However, it is well known that severe side effects, for example, nephrotoxic, ototoxic, and neurotoxic effects are associated with cisplatin therapy. It has been shown that cisplatin strongly interferes with ATP-dependent cation translocation by SR Ca^2+^-ATPase and Na^+^,K^+^-ATPase [53], which have been proposed as potential cisplatin targets. The SSM measurements indicated that cisplatin inhibition of SR Ca^2+^-ATPase activity is stronger (IC_50_ = 1.3 µM, Figure 4) than that observed in the case of Na^+^,K^+^-ATPase (IC_50_ = 11.1 µM). Therefore, cisplatin inhibition of the transport activities of these two enzymes could be relevant to the mechanisms underlying the different side effects of cisplatin.

Development of cell resistance to cisplatin-based therapies represents a critical issue that considerably reduces the efficacy of platinum anticancer drugs. Interaction of cisplatin with mammalian Cu^+^-ATPases, ATP7A and ATP7B, also known as Menkes and Wilson disease proteins, has been associated with resistance of cancer cells to platinum drugs [55,56,57]. ATP7A and ATP7B, which are localized in the trans-Golgi network (TNG), perform active transfer of copper across the membrane into the TGN lumen by ATP utilization and are responsible for regulating intracellular copper levels [58]. It was reported that cisplatin is a substrate for ATP7B and the enzyme can translocate cisplatin at a slower rate than copper [59]. In order to gain insights, at a molecular level, into translocation of platinum drugs by Cu^+^-ATPases we employed the SSM method to investigate the mechanism of interaction of cisplatin and oxaliplatin, a third-generation platinum analogue that is active in patients with colorectal cancer, with human ATP7A and ATP7B [60]. SSM measurements on vesicles containing ATP7A or ATP7B indicated that cisplatin and oxaliplatin activate the ATPase cycle and, in the presence of ATP, can be translocated across the vesicle membrane. NMR spectroscopy and ESI-MS were used to determine the binding mode of these platinum drugs to the ATP7A amino-terminal extension [60,61]. It was suggested that translocation of platinum drugs by ATP7A and ATP7B and sequestration of these drugs in the ATPase amino-terminal extension are likely to contribute to drug resistance of cancer cells.

The SSM method was also employed to investigate the interaction of anticancer ruthenium-based compounds, i.e., NAMI-A, RAPTA-C, and KP1019, with SR Ca^2+^-ATPase [62]. Preclinical studies showed that NAMI-A could act as an effective antimetastatic drug, whereas KP1019 was found to be active against colorectal cancers [63]. The SSM measurements indicated that KP1019, in contrast to the other Ru(III) complexes, was capable of interfering strongly with SR Ca^2+^-ATPase function. In particular, an IC_50_ value of 1 µM was determined for inhibition of calcium translocation by KP1019. It was hypothesized that KP1019 interaction with SR Ca^2+^-ATPase determines uncoupling of ATP hydrolysis with transport of Ca^2+^ ions, thereby decreasing calcium translocation across the SR membrane.

The examples described above (see Table 2) demonstrate the usefulness of the SSM technique to investigate the activity and mode of action of anticancer drugs that are targeted against various membrane transporters, and qualify the SSM method as a robust, flexible, and reliable assay for drug screening applications.

## 4. Electrochemical Nucleic Acid-Based Sensors

The interaction of anticancer drugs with DNA is among one of the most important aspects of biological studies in drug discovery and pharmaceutical development processes.

**Figure 5:**
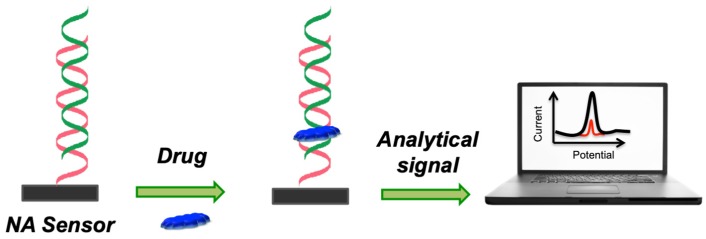
Scheme of a nucleic acid-based sensor. The change in the oxidation signal of guanine or adenine is frequently monitored as a consequence of the interaction of the drug with the nucleic acid strands.

Electrochemical nucleic acid-based sensors are other bioelectrochemical platforms for studying the interaction of drugs with DNA. An electrochemical nucleic acid-based sensor is defined as a device that integrates nucleic acids (natural and biomimetic forms of oligo- and polynucleotides) as the biological recognition element and an electrode as the physicochemical transducer [64]. According to the biomolecular interactions, nucleic acid-based sensors can be classified as affinity biosensors (i.e., genosensors [65,66,67] and aptasensors [68]), catalytic biosensors (i.e., aptazyme-based sensors), and nucleic acid-based sensors for monitoring of chemically-induced DNA structure modification [69,70,71]. The latter configuration allows the evaluation of DNA–molecule interactions and DNA damage assessment [72,73,74]. Small molecules, including drugs, can interact with DNA in different modes, classified as a noncovalent association, i.e., electrostatic interactions, binding at major or minor grooves of the DNA double helix and intercalation between the stacked base pairs of double stranded DNA (ds DNA) [75]. Some other compounds (such as mitomycin C) form covalent bonds with nucleic acid bases to create adducts. Synthetic oligonucleotides, genomic DNA [69,76], or stem loop structures [77] can be used to assess the alterations induced by the molecule on DNA. Altered structural, chemical, and physicochemical properties of DNA are reflected in its behavior at the electrochemical transducer, since the binding of drug molecules to DNA causes a change in the intrinsic electrochemical signal of the DNA, i.e., adenine and guanine redox signals (Figure 5). Thus, this kind of DNA-electrochemical biosensor directly monitors the changes in the DNA bases oxidation peaks before and after the interaction with the drug and it can be classified as a label-free biosensor. Moreover, this kind of biosensor has also been proposed to monitor the level of anticancer drugs in biological fluids.

The biomolecular interactions between DNA and the anticancer drug dacarbazine (DCB), a molecule frequently used for the treatment of metastatic malignant melanoma, was investigated using a single-walled carbon nanotube modified disposable pencil graphite electrode [78]. DCB acts as alkylating agent and its binding to DNA strands affects the intrinsic electrochemical activity of DNA; moreover, it is an electroactive molecule itself. Thus, the oxidation signals of both DCB and guanine can be measured using differential pulse voltammetry. The voltammetric results reported in [78] were found in good agreement with gel electrophoresis analysis. A decrease of ethidium bromide (EtBr) luminescence intensity was observed in contrast to the control in PCR samples while increasing the incubation time of DCB. The binding of DCB to DNA was claimed to impede EtBr binding. Furthermore, this DNA sensor was proposed to be a sensitive method for DCB determination in urine or other biological fluids with a detection limit of 1.1 μM within 5 min.

**Figure 6:**
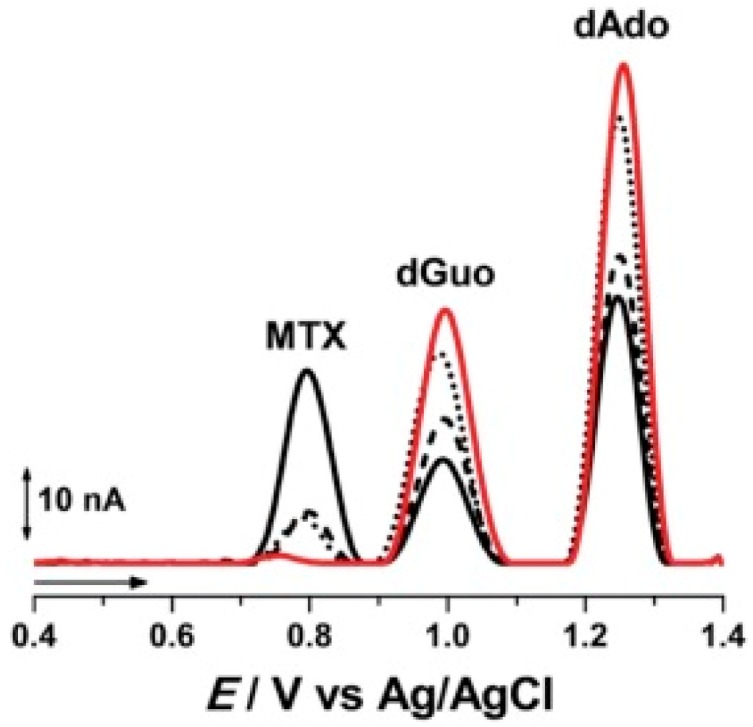
Examples of voltammetric oxidation peaks recorded in pH 4.5, 0.1 M acetate buffer with electrochemical nucleic acid-based sensors (red line) before and after incubation during (black line) 5, (▪ ▪ ▪) 10, and (•••) 20 min in a solution of 100 µM Methotrexate (MTX). (from [80] with permission).

Methotrexate (MTX) is one of the earliest anticancer drugs used in some types of leukemia, lung cancer, sarcoma, etc. MTX is classified as an antimetabolite cancer drug because it targets the enzyme dehydrofolate reductase, responsible for folic acid production, and plays a supporting, but essential, role for the synthesis of thymine nucleotide [79]. MTX treatment causes an accumulation of 8-oxoG in cells. Pontinha et al. showed that the interaction of MTX with DNA leads to modifications to the DNA structure in a time-dependent manner [80]. The DNA–MTX interaction was evaluated by AFM at a highly oriented pyrolytic graphite surface and by voltammetry using a nucleic acid-based sensor. The intercalation of MTX in DNA led to ds DNA unwinding, as shown by the increase of the purine residues oxidation peaks reported in Figure 6, confirmed by AFM micrographs showing a reorganization of the DNA self-assembled network upon MTX binding. More recently, a graphene oxide modified glassy carbon electrode (DNA/GO/GCE) was used to develop an electrochemical sensor for monitoring MTX–DNA interactions via guanine oxidation [81]. The DNA/GO/GCE sensor showed a detection limit of 7.6 nM and was tested for MTX determination in spiked urine and blood serum samples.

The interactions of EGFR exon 21-point mutant gene with the anticancer drug Gemcitabine was recently evaluated using a DNA biosensor as reported in [82] and summarized in Table 3. Gemcitabine is one of the most important therapeutic agents of early and advanced stages of non-small cell lung cancer. The treatment occurs by activating EGFR mutations, especially the L858R point mutation and exon 19 deletions. For this reason, the point mutation (L858R) sequence of EGFR exon 21 and its complementary single-stranded DNA was selected to form the double helix structure as a bioreceptor for developing the biosensor. EGFR exon 21 acts as an identification probe but also as an electrochemical indicator via direct monitoring of guanine and adenine oxidation signal before and after the interaction with the drug. The oxidation signals of adenine and guanine were in a linear range when the device was subjected to various concentrations of Gemcitabine, from 1.5 to 93 μM, where detection limits of 12.5 nM, and 48.8 nM were recorded by guanine and adenine respectively.

## 5. Other Bioelectrochemical Approaches

Many anticancer drugs interact with redox machinery of the cell and cellular homeostasis including reactive oxygen or nitrogen species (ROS, RNS) [83]. Indeed, drugs interact with many other different metabolites and cellular components increasing or lowering their concentration. Electrochemistry offers many interesting solutions for the monitoring of these metabolites [84], including fast voltammetry and amperometry at micro- and nanoelectrodes [83,85,86], chip-based electrochemical platforms [87,88,89,90], SECM/SICM configuration for cell imaging and protein, nucleic acid analysis [90,91], electrochemical biosensor technology [92,93,94].

An interesting area is the study and simulation of drug metabolism coupling electrochemistry with mass spectrometry (MS) [95,96]. Electrochemistry coupled to MS can provide increased throughput and information on short-lived species. To this end, we ask the reader to refer to a recent review [97] for a description of the metabolism of various substances in the human body and for a summary of methods used for prediction of metabolic pathways and biotransformation, with special emphasis on the coupling of electrochemistry to MS.

Finally, among the different bioelectrochemical approaches, it is worth mentioning that organic bioelectronics [98,99,100,101,102] is receiving great attention for its potential application in real-time selective noninvasive detection of chemical biomarkers, including drugs, metabolites, neurotransmitters, proteins, and hormones, in a variety of body fluids.

## 6. Conclusions

New anticancer drugs with increased effectiveness, less toxicity, and limited side effects are needed. To address these pharmaceutical needs, great effort is currently devoted to the development of multiplexed, reliable, and rapid screening methods to analyze the mode of action of anticancer drugs and to identify novel drug candidates. In this review, we have discussed different label-free bioelectrochemical methods that can be conveniently used for the analysis of anticancer drug interactions with nucleic acids, membrane proteins, and living cells. If cell impedance-based methods present the features for high-throughput drug screening analysis, SSM- and DNA-based methods in a medium- to low-throughput approach can be used to evaluate the interaction between the anticancer drug and the target at a molecular level. Such methods provide robust, flexible, and reliable assays and have potential for the implementation of simple and cost-effective analytical tools for drug screening applications.

## Figures and Tables

**Table 2:** Interactions of anticancer drugs with P-type ATPases studied by the SSM technique.

Anticancer Drug	Observed Effect	Specific Comments	Ref
Cisplatin	Inhibition of Na^+^,K^+^-ATPase and SR Ca^2+^-ATPase	Strong and irreversible inhibition of SR Ca^2+^-ATPase activity. Reversible inhibition of Na^+^,K^+^-ATPase activity.	[53]
Cisplatin and Oxaliplatin	Translocation by Cu^+^-ATPases (ATP7A and ATP7B)	Binding and translocation of Pt-drugs across the vesicle membrane.	[60]
NAMI-A, RAPTA-C and KP1019	Inhibition of SR Ca^2+^-ATPase	Strong inhibition of Ca^2+^ translocation by SR Ca^2+^-ATPase.	[62]

**Table 3:** Some examples of anticancer drugs studied by nucleic acid-based sensors.

Anticancer Drug	Observed Effect	Specific Comments	Ref
Dacarbazine	Guanine oxidation signal	Analysis of PCR amplicons and comparison with Gel electrophoresis	[78]
Methotrexate	Guanine oxidation signal and MTX oxidation signal	Analysis of spiked serum samples and urine samples	[81]
Gemcitabine	Guanine and adenine oxidation signal	Analysis of spiked serum samples	[82]
